# Development of refractive error in children treated for retinopathy of prematurity with anti-vascular endothelial growth factor (anti-VEGF) agents: A meta-analysis and systematic review

**DOI:** 10.1371/journal.pone.0225643

**Published:** 2019-12-02

**Authors:** Qing-Qing Tan, Stephen P. Christiansen, Jingyun Wang

**Affiliations:** 1 Department of Ophthalmology, Affiliated Hospital of North Sichuan Medical College, Nanchong, Sichuan, China; 2 Department of Ophthalmology and Optometry, North Sichuan Medical College, Nanchong, Sichuan, China; 3 Salus University Pennsylvania College of Optometry, Elkins Park, Pennsylvania, United States of America; 4 Department of Ophthalmology, Boston University School of Medicine, Boston, Massachusetts, United States of America; 5 Department of Pediatrics, Boston University School of Medicine, Boston, Massachusetts, United States of America; 6 Boston Medical Center, Boston, Massachusetts, United States of America; National Yang-Ming University Hospital, TAIWAN

## Abstract

**Objective:**

To investigate refractive error development in preterm children with severe retinopathy of prematurity (ROP) treated with anti-vascular endothelial growth factor (anti-VEGF) agents and laser photocoagulation.

**Methods:**

Selection criteria were comparative studies that compared the refractive errors in children, birthweights ≤1500 grams and gestational ages ≤30 weeks, and treatments for Type I ROP with intravitreal bevacizumab (IVB) versus laser photocoagulation. Studies were identified using PubMed, Google Scholar, and published reviews. Meta-analyses were performed on the post-treatment outcomes of spherical equivalent (SEQ), cylindrical power, and prevalence of high myopia. Longitudinal development of refractive error in IVB, or in laser-treated children, or in normal full-term children was visually summarized.

**Results:**

Two randomized controlled trials and 5 non-randomized studies, including a total of 272 eyes treated by IVB and 247 eyes treated by laser, were included in this study. Compared with laser-treated children, IVB-treated children have less myopic refractive error (*P*<0.001), lower prevalence of high myopia (*P*<0.05), and less astigmatism (*P* = 0.02).

**Conclusions:**

Treatment with IVB is associated with less myopia and astigmatism than laser treatment for infants with severe ROP. Given the complexity of ROP and the variability of dosing, our review supports close monitoring of refractive error outcomes in children treated with IVB.

## Introduction

For three decades, children with severe retinopathy of prematurity (ROP) have been treated by laser photocoagulation. Laser-treated children had a high incidence of myopia or high myopia [1–3]. The Early Treatment of Retinopathy of Prematurity (ETROP) study found that, at 3 years of age, the prevalence of myopia in infants with severe ROP was 65–71% and the prevalence of high myopia (≤-5.00D) was 51% [2]. It has been suggested that the mechanism for ROP-related myopia is different than that for common myopia which develops in school-age children[4]. In a longitudinal study, we found that myopia associated with severe ROP develops earlier in life, often before 1.5 years of age [5].

More recently, because vascular endothelial growth factor (VEGF) is a key factor in the progression of ROP, anti-VEGF agents have been used as a treatment modality. Currently, two anti-VEGF agents have been studied: intravitreal bevacizumab (IVB) and intravitreal ranibizumab (IVR). Bevacizumab is more frequently used than ranibizumab due to its lower cost [6]. In this study, we specifically evaluate refractive error development in IVB-treated infants.

### Complexity of investigating refractive error in IVB-treated children with ROP

Studies from different institutions and different countries have reported post-IVB refractive errors. It is important to understand that there are several variables, which differ amongst studies that preclude the direct comparison of refractive error results across the studies. Some of these variables are as follows:

#### Gestational age and birth weight

While infants treated in US or Europe were, on average, 24~25 weeks [7–11] and those in Taiwan were, on average, 26~27 weeks [12–15], patients treated in Mexico were 27–32 weeks of gestational age [16]. It was suggested that retina development is highly related to a gestational age of 24 to 28 weeks. Earlier gestational age and lower birth weight are highly associated with more severe prematurity. Therefore, refractive outcomes could vary among studies, countries and areas.

#### Severity of ROP

Given the same treatment, patients with different zones and stages may respond differently and thus have different outcomes. While IVB is generally applied to both eyes, sometimes IVB treatment is applied to only one eye. For severe cases, physicians may combine both laser and IVB treatments. Yoon et al. combined IVB with laser or IVB with deferred laser to treat Zone I ROP, and they reported that IVB with deferred laser treatment had less myopia [17]. Therefore, when comparing IVB-treated with laser-treated preterm children, it is more convincing if the study is randomized. In this analysis, we include those study outcomes that result from a single treatment modality.

#### Refraction age and duration of follow-up

So far, most studies on IVB treatment reported cross-sectional refractive error outcome at approximately 1 year of age to 3 years of age [9, 10, 12–15, 18–27]. Only one study reported longitudinal data up to 5 years of age [16].

#### Methods of measuring refractive error

Many institutions used cycloplegic retinoscopy to measure refractive error [10, 18], while some studies used an autorefractor under cycloplegia [9, 13, 14]. For instance, the Gunay et al. study used the SureSight autorefractor to measure refraction with cycloplegia[22]. Although the SureSight autorefractor is an effective instrument for screening, it has significant limitations of the range of refractive error, especially astigmatism [28–30]. Therefore, their results must be interpreted with caution.

#### Recurrence rate of ROP after anti-VEGF treatment

Recurrence rates ranging from 6% [31] to 14% [10] could be an important factor. Generally, patients who had recurrence in the laser-treated group were more myopic [2, 32]. IVB seems to be associated with low recurrence rates and ocular complication rates [33]. BEAT-ROP reported the refraction in patients who had recurrence separately; there were only 2 eyes in both Zone I and Zone II subgroups for IVB groups. From limited data, in eyes treated for recurrence of ROP with additional IVB, there was a further increase in myopia. But it is hard to conclude for IVB groups so far [7].

#### Other factors

Another factor is IVB dosage. Although generally a 0.625mg (0.024mL) IVB dosage was used, some studies used a higher dosage of 1.25 mg [16] or a lower dosage of 0.375mg [8, 9] or 0.5mg [14]. Injection sites vary between 1mm to 2mm behind the limbus; more posterior injections are associated with increased risk of retinal detachment [34] (ARVO abstract). Unfortunately, few studies reported their injection site and we are not able to further review this factor.

Several excellent reviews have investigated refractive error, safety, and efficacy of IVB compared with laser therapy. Li et al. suggested that laser treatment might be more efficacious than anti-VEGF but result in more eye complications and higher myopia [35]. Mintz-Hittner & Geloneck also suggested that anti-VEGF agents may result in less severe myopia [36]. Both Sankar et al. and Abri Aghdam et al. agreed with this conclusion [37, 38]. These studies summarized refractive error outcomes in SEQ with different follow-up periods ranging from 1 to 3 years. Despite this, predicting individual refractive error development remains highly imprecise [38]. In addition, there is little information related to astigmatism even though it is important for children with ROP [2, 32].

In this review, combined with meta-analysis of published data, we present a systematic evaluation of risk for myopia and astigmatism following monotherapy of IVB versus laser treatment for Type I ROP. The goals of this review are 1) to compare the magnitude and range of myopia in children treated with IVB and those with laser; 2) to compare the prevalence of high myopia (SEQ≤ -5.00D) in children treated with IVB and those with laser; 3) to compare the magnitude of astigmatism in children treated with IVB and those with laser; and, 4) to summarize and plot longitudinal development of SEQ refractive error in these children treated with IVB.

## Materials and methods

The present meta-analysis was conducted according to the Preferred Reporting Items for Systematic Reviews and Meta-Analyses (PRISMA) statement [39].

### Literature search and study selection criteria

An extensive search of the peer-reviewed literature was completed using PubMed and Google Scholar with the following search strategy: (“ROP” OR “Retinopathy of prematurity”) AND (“anti-VEGF” OR "anti-vascular endothelial growth factor" OR “intravitreal IVB” OR “IVB”). The search was performed by two authors (QT and JW) during August of 2018 and March of 2019.

Selection criteria were: 1) study participants were preterm children with birthweights ≤1500 grams and gestational ages ≤30 weeks, who met criteria for ROP screening examination established by the American Academy of Pediatrics [40]; 2) participants were diagnosed with type I ROP, which was defined by the ETROP study as Zone I with any stage with plus disease, Zone I with stage 3 without plus disease, and Zone II with stage 2 or 3 with plus disease [41]; 3) comparative studies of IVB versus laser photocoagulation treatments for Type I ROP; and, 4) post-treatment refractive error data was followed up as one of the study outcomes.

### Data extraction and quality assessment

For each included study, the first author, publication year, name of the journal, country, study design, sample size, gestational age, birthweight, follow-up time, agent for refraction, refraction methods, dose of IVB, means and standard deviations (SD) of SEQ and cylindrical power were extracted. We also extracted the prevalence of high myopia. The quality of the RCTs was evaluated using the Cochrane risk of bias tool [42]. The following parameters were considered: 1) random sequence generation; 2) allocation concealment; 3) blinding of participants and personnel; 4) blinding of outcome assessment; 5) incomplete outcome data addressed; 6) selective reporting; 7) other bias. Each of the parameters was graded as: Low risk of bias, High risk of bias, or Unclear risk of bias. The quality of the NRSs was evaluated using the Newcastle-Ottawa Scale (NOS) [43]. The NOS scale consists of a total of 8 items in 3 categories: Selection (4 items); Comparability (1 item); Exposure (3 items). A study can be awarded a maximum of one star for each numbered item within the Selection and Exposure categories. A maximum of two stars can be given for the Comparability category. NOS stars of 0–3, 4–6, and 7–9 were considered to indicate low, moderate and high quality, respectively.

### Statistical analysis

Meta-analysis was performed in subgroups of randomized controlled trials (RCTs) and non-randomized studies (NRSs) respectively using Review Manager analysis software (RevMan 5.3, The Cochrane Collaboration, 2014). Means and standard deviations of SEQ and cylindrical power were pooled for all the available data. Mean differences were calculated with 95% confidence interval (CI). Based on the number of eyes presenting with high myopia after treatment, risk ratios and odds ratios were calculated with 95% CI to estimate the effects for prevalence of high myopia for RCTs and NRSs respectively. A random effects model was used to calculate pooled estimates. Heterogeneity between the studies was tested using the *I*^*2*^ statistic, with *I*^*2*^ values over 50% indicating significant heterogeneity. When significant heterogeneity presented, sensitivity analysis was performed by omitting individual studies to assess the robustness of meta-analysis results.

## Results

### Literature search results and study characteristics

**Fig 1:**
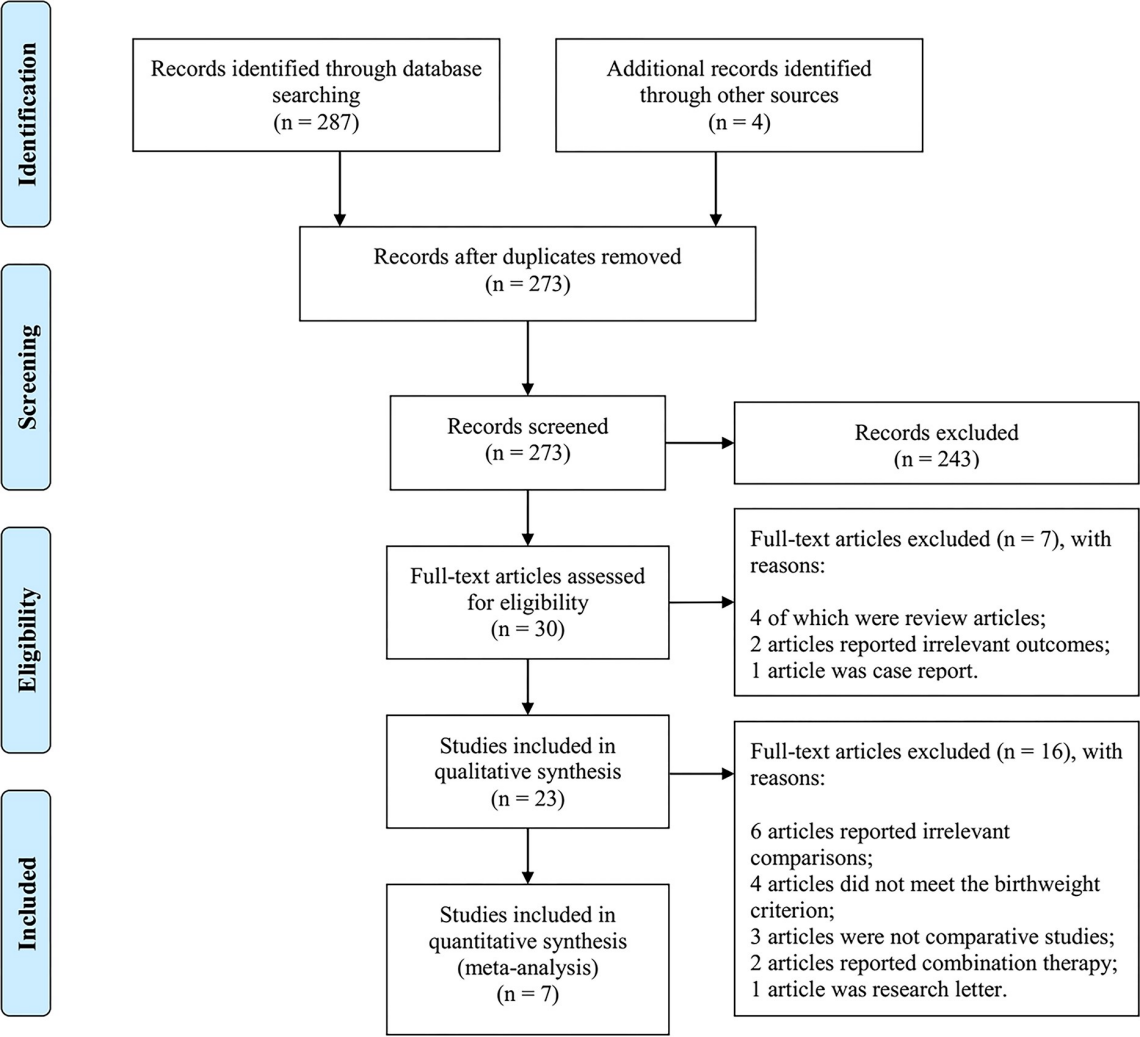
PRISMA flow diagram of study selection.

As shown in **Fig 1**, after duplicates were removed, 273 records were screened. A total of 30 full-text articles were assessed for eligibility according to the titles and abstracts. Twenty-three relevant studies were included for a qualitative synthesis by table and plot. Two RCTs (Geloneck 2014 [44]; O'Keeffe 2016 [45]) and 5 NRSs (Harder 2013 [8]; Gunay 2015 [22]; Hwang 2015 [10]; Isaac 2015 [11]; Lee 2018 [46]), including a total of 272 eyes treated by IVB and 247 eyes treated by laser, were ultimately included for a quantitative synthesis by meta-analysis. Two of 7 studies implemented comparisons in Zone I and Zone II ROP classifications [10, 44]. Therefore, 9 pairs of data were eventually pooled. In all 7 studies, the mean gestational ages ranged from 24.3 to 26.6 weeks, and the mean birth weights ranged from 625 to 901 grams. **Table 1** summarizes the literature that reports refractive error data in children treated with IVB.

**Table 1:** Summary of IVB-treated ROP literature.

Study	Study design	Intervention	No. eyes	Mean refraction age (yr)	Mean GA (wk)	Mean BW (g)	Mean SEQ (range, D), *P* value	Prevalence of high myopia^¶^	Mean astigmatism (range, D), *P* value	Anti-VEGF dose (mg)
*Meta-analysis included studies*										
Geloneck 2014[44]	RCT	IVB vs. Laser	110 vs. 101	2.5	Zone ǀ: 24.3; Zone ǁ: 24.4	Zone ǀ: 625; Zone ǁ: 625	Zone ǀ: -1.51 (-8.56 to 6) vs. -8.44 (-24.88 to 2), *P*<0.001; Zone ǁ: -0.58 (-13 to 2.5) vs. -5.83 (-19 to 3.5), *P*<0.001	Zone ǀ: 21% vs. 54%; Zone ǁ: 5% vs. 49%	—	0.625
O'Keeffe 2016[45]	RCT	IVB vs. Laser	15 vs. 15	5	25	780	-0.9 (-8 to 2) vs. -2.73 (-12 to 2)	—	—	1.25
Harder 2013[8]	NRS	IVB vs. Laser	23 vs. 26	0.9	25.3	450 to 1115	-1.04 (-12.5 to 4.63) vs. -4.41 (-14 to 4.38), *P* = 0.02	17% vs. 54%	1.0 (0 to 5) vs. 1.82 (0 to 6), *P* = 0.03	0.375 or 0.625
Hwang 2015[10]	NRS	IVB vs. Laser	20 vs. 29	1.9 vs. 3.1	Zone ǀ: 24.3; Zone ǁ: 24	Zone ǀ: 668; Zone ǁ: 669	Zone ǀ: -3.7 (-8.9 to 0.3) vs. -10.1 (-16.5 to 2), *P* = 0.41; Zone ǁ: 0.6 (-1.1 to 2.5) vs. -4.7 (-16 to 0), *P* = 0.002	—	Zone ǀ: 1.2 (0 to 2.5) vs. 2.1 (1 to 3.25), *P* = 0.13; Zone ǁ: 0.6 (0 to 1.75) vs. 1.6 (0 to 5), *P* = 0.19	0.625
Gunay 2015[22]	NRS	IVB vs. Laser	48 vs. 30	2	26.4	901	0.42 (-8.75 to 5) vs. -6.66 (-15.5 to 1.75), *P* = 0.001	8% vs. 73%	—	0.625
Isaac 2015[11]	NRS	IVB vs. Laser	23 vs. 22	0.9	25.2 vs. 25	722 vs. 674	-3.57 (-15 to 6.5) vs. -6.39 (-13 to 0.5), *P* = 0.33	35% vs. 59%	—	0.625
Lee 2018[46]	NRS	IVB vs. Laser	33 vs. 24	4.8 vs. 4.9	26.6 vs. 26.6	874 vs. 803	-0.1 vs. -2.5, *P* = 0.003	—	1.3 vs. 1.4, *P* = 0.14	0.625
*Studies also included in* ***our figure***										
Martinez-Castellanos 2013[16]	Case series	IVB	9	5	29.3	850 to 1600	-1.75 (-6.75 to 2.5)	11%	—	1.25
Wu 2013[15]	Case series	IVB	53	1	26.3	930	-0.1 (-8.75 to 6.55)	8%	2.1 (0.3 to 5.3)	0.625
Chen 2014[12]	NRS	IVB vs. (IVB+ Laser) vs. (IVB+LSV)	40 vs. 17 vs. 7	2	26.6 vs. 24.7 vs. 28.6	879 vs. 732 vs. 1164	-0.98 (-15.6 to 5.5) vs. -2.4 (-7.6 to 2.9) vs. -14.38 (-21.8 to -8.1), *P*<0.001	10% vs. 29.4% vs. 100%	2.23 (0.3 to 6.8) vs. 2.32 (0.5 to 4.8) vs. 3.11 (1.5 to 6), *P* = 0.291	0.625
Kuo 2015[14]	NRS	IVB vs. Laser	30 vs. 28	3	27.3	1080 vs.1006	-1.53 (-5.88 to 1.5) vs. -1.71 (-4.38 to 0.13), *P* = 1	0% vs. 0%	—	0.5
Chen 2015[13]	NRS	IVB	41	1	26.5	869	-0.3 (-1.6 to 1.1)	14.6%	1.8 (1.5 to 2.2)	0.625
Araz-Ersan 2015[19]	NRS	(IVB+Laser) vs. Laser	18 vs. 13	2	27.3 vs. 27.7	1017 vs. 988	-0.15 vs. 1.43, *P* = 0.16	—	1.26 vs. 0.57, *P* = 0.35	0.625
Lin 2016[26]	NRS	IVB	15	1	26.5	938	-0.6 (-10.63 to 4.5)	—	—	0.625
Gunay 2017[23]	NRS	IVB vs. Laser	55 vs. 57	1.5	27.3 vs. 28.2	1005 vs.1119	-0.57 vs. -0.81, *P* = 0.13	12.7% vs. 14%	—	0.625
Kabatas 2017[24]	NRS	IVB vs. Laser	24 vs. 72	1.5	26.1 vs. 27.7	841 vs. 1112	-1.49 vs. -1.27, *P* = 1	—	1.31 vs. 1.75, *P* = 0.151	0.625
Kimyon 2018[25]	NRS	IVB	40	1	29.3	1361	-1.49	12.5%	—	0.625
Roohipoor 2018[27]	NRS	IVB vs. Laser	397 vs. 190	2.3 vs. 2	27.8	1146	-1.26 vs. -2.84, *P* = 0.016	—	1.79 vs. 1.84	0.625
Chen 2018[21]	NRS	IVB	36	3	27.1	911	-0.65 (-0.17 to 1.09)	16.7%	1.6	0.625
*Other referred studies*										
Axer-Siegel 2011[20]	Case series	IVB	10	0.9 to 1.5	24 to 26	620 to 825	-5 to 6	20%	—	0.625
Harder 2012[9]	NRS	IVB vs. Laser	12 vs. 20	0.9	24.8	480–810	RE: -0.27 (-7 to 4.25) vs. -6.25 (-12 to 3.5), *P* = 0.03; LE: 1.54 (-0.75 to 4.63) vs. -4.2 (-14 to 4.38), *P* = 0.02	—	RE: 1.13 (1 to 2) vs. 1.80 (0 to 5), *P* = 0.22; LE: 0.92 (0 to 2) vs. 1.58 (1 to 3), *P* = 0.09	0.375
Kang 2019 [47]	NRS	(IVB/IVR) vs. Laser	22 vs. 30	4	27.4 vs. 34	983.2 vs. 961	Zone ǀ: -1.22 vs. -2.69; Zone ǁ: -0.32 vs. -1, *P* = 0.603	—	Zone ǀ: 0.69 vs. 3.88; Zone ǁ: 0.32 vs. 1, *P* = 0.294	0.625

GA: gestational age; BW: birth weight; yr: years; wk: weeks; SEQ: spherical equivalent; D: diopter; RCT: randomized controlled trial; NRS: non-randomized controlled study; IVB: intravitreal bevacizumab; IVR: intravitreal ranibizumab; LSV: lens-sparing vitrectomy; RE: right eyes; LE: left eyes; ^¶^: myopia≤-5.00D.

### Study quality assessment

**Fig 2:**
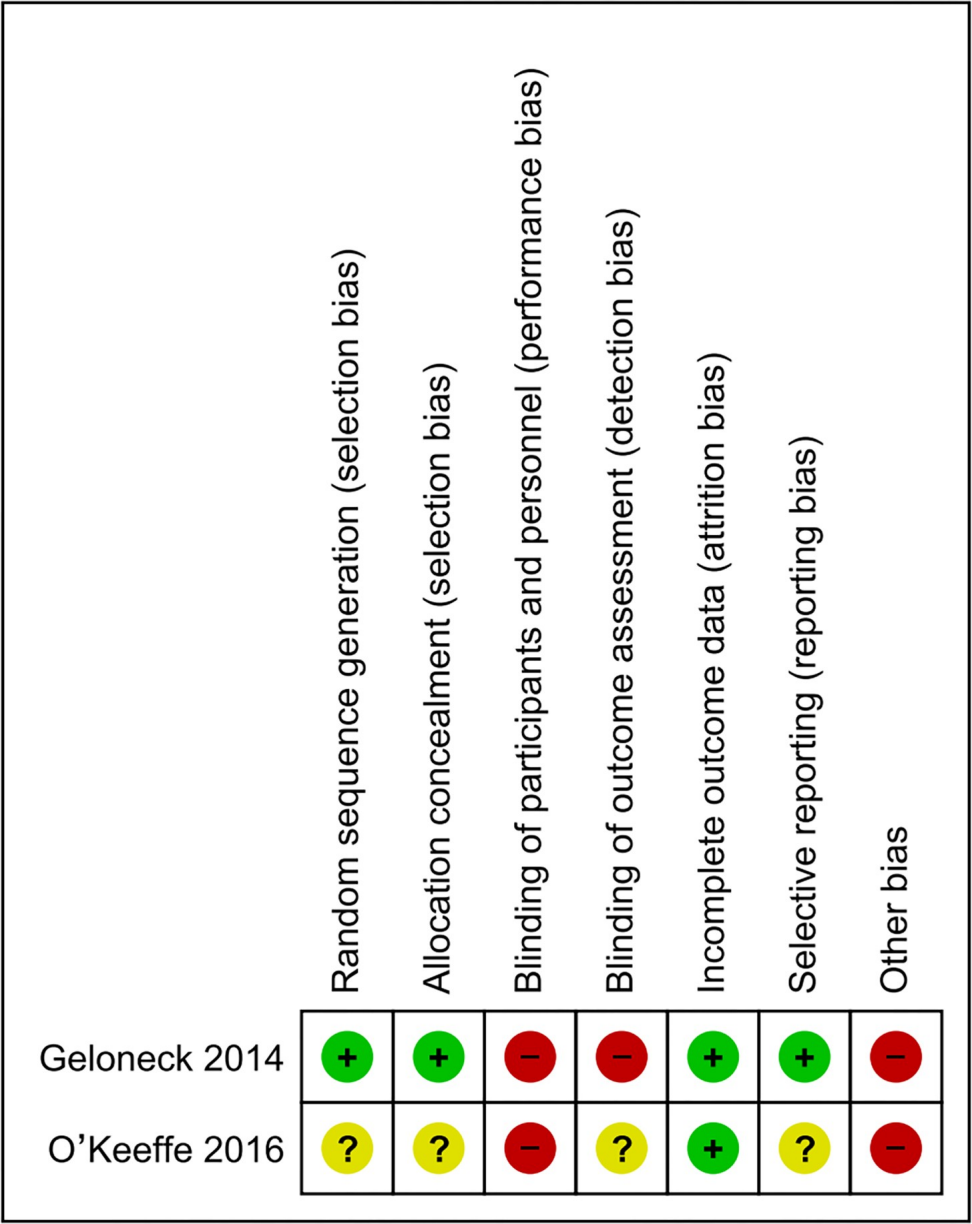
Risk of bias summary for included RCTs.

The quality assessment of 2 RCTs is shown in **Fig 2**. Overall, the Geloneck et al. study [44] was judged to have low risk of bias and the O’Keeffe et al. study [45] was judged to have high risk of bias. The quality assessment of 5 NRSs is shown in **Table 2**. All 5 NRSs were scored greater or equal to 7, which indicated high quality.

**Table 2:** Quality assessment of included NRSs using NOS.

Study	Harder 2013	Gunay 2015	Hwang 2015	Isaac 2015	Lee 2018
Is the case definition adequate?	★	★	★	★	★
Representativeness of the cases	★	0	★	★	0
Selection of controls	0	0	★	0	0
Definition of controls	★	★	★	★	★
Comparability of cases and controls on the basis of the design or analysis^§^	★★	★★	★★	★★	★★
Ascertainment of exposure	★	★	★	★	★
Same method of ascertainment for cases and controls	★	★	★	★	★
Nonresponse rate	★	★	0	0	★
Total scores	8	7	8	7	7

NOS: Newcastle-Ottawa Scale;

★ indicates a score of 1;

★★ indicates a score of 2;

^§^: The marked category has a maximum score of 2.

### Magnitude of myopia in children treated with IVB versus those treated with laser

The mean SEQ ranged from -3.7 ± 3.3 diopters (D) to 0.6 ± 1.7D in IVB treatment and

**Fig 3:**
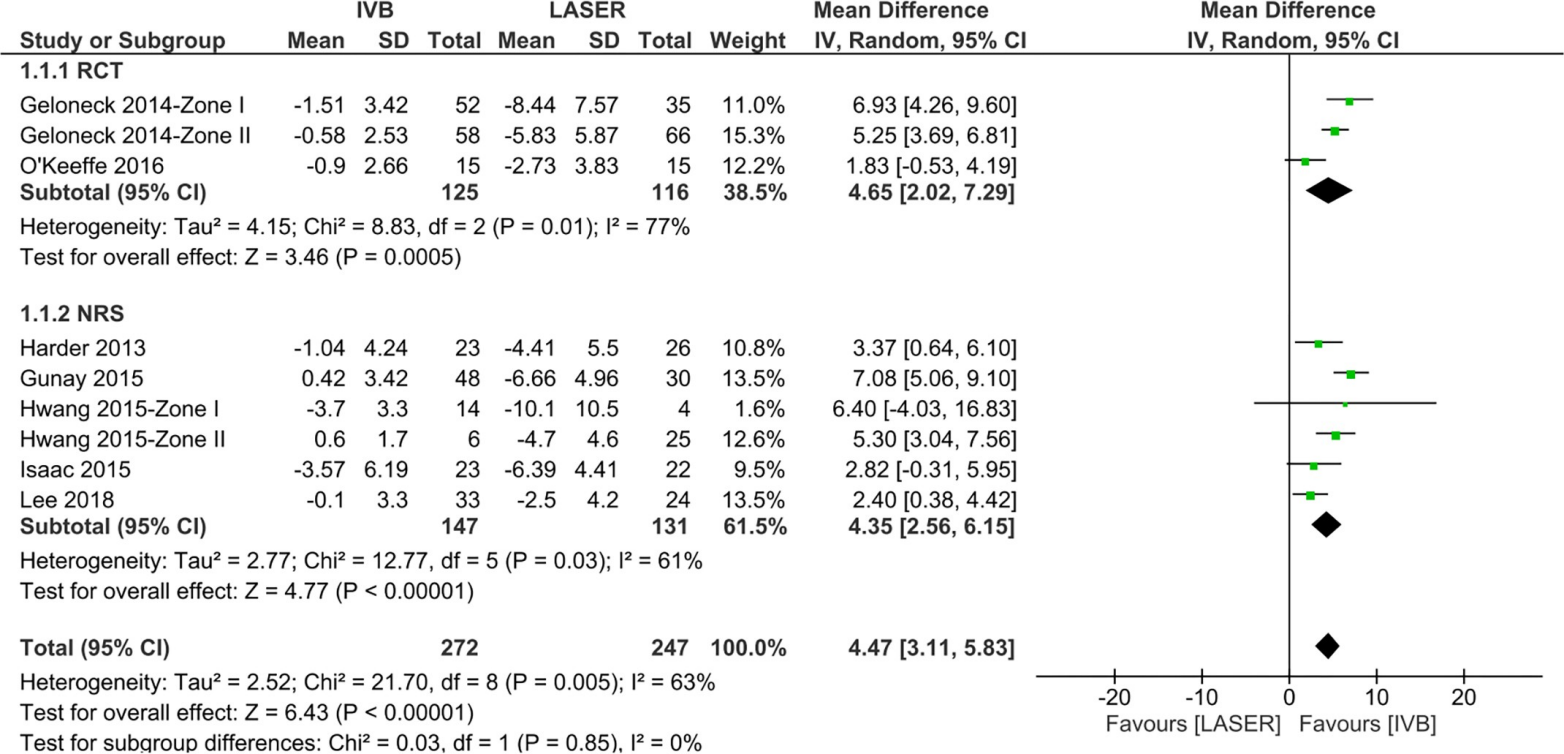
Forest plot of the comparison of SEQ between IVB and LASER treatments. Pooled estimates for mean differences and 95% CI in diopters between two treatments. The black diamond symbol shows the estimated true effect size.

-10.1 ± 10.5D to -2.5 ± 4.2D in laser treatment. A statistically significant lower myopic SEQ was shown in IVB treatment in both subgroups (RCT: mean difference = 4.65D; 95% CI, 2.02 to 7.29; *P*<0.001; NRS: mean difference = 4.35D; 95% CI, 2.56 to 6.15; *P*<0.001), with significant heterogeneities in both RCTs (*I*^*2*^ = 77%, *P* = 0.01) and NRSs (*I*^*2*^ = 61%, *P* = 0.03). The high heterogeneity in RCTs was essentially due to the high risk of bias in the O’Keeffe et al. study [45], and the difference in the follow-up age (Geloneck 2014 [44]: 2.5 years; O’Keeffe 2016: 5 years) and IVB dose (0.625mg vs. 1.25mg) between studies. By omitting the O’Keeffe et al. study, heterogeneity was reduced from 77% to 12% and the overall effect was not inversed. For NRSs, by omitting the Gunay et al. study, heterogeneity was reduced from 61% to 1% and the overall effect was not inversed as well. Sensitivity analyses indicated robust meta-analysis results. (**Fig 3**)

### Prevalence of high myopia (SEQ≤ -5.00D) in children treated with IVB versus those treated with laser

**Fig 4:**
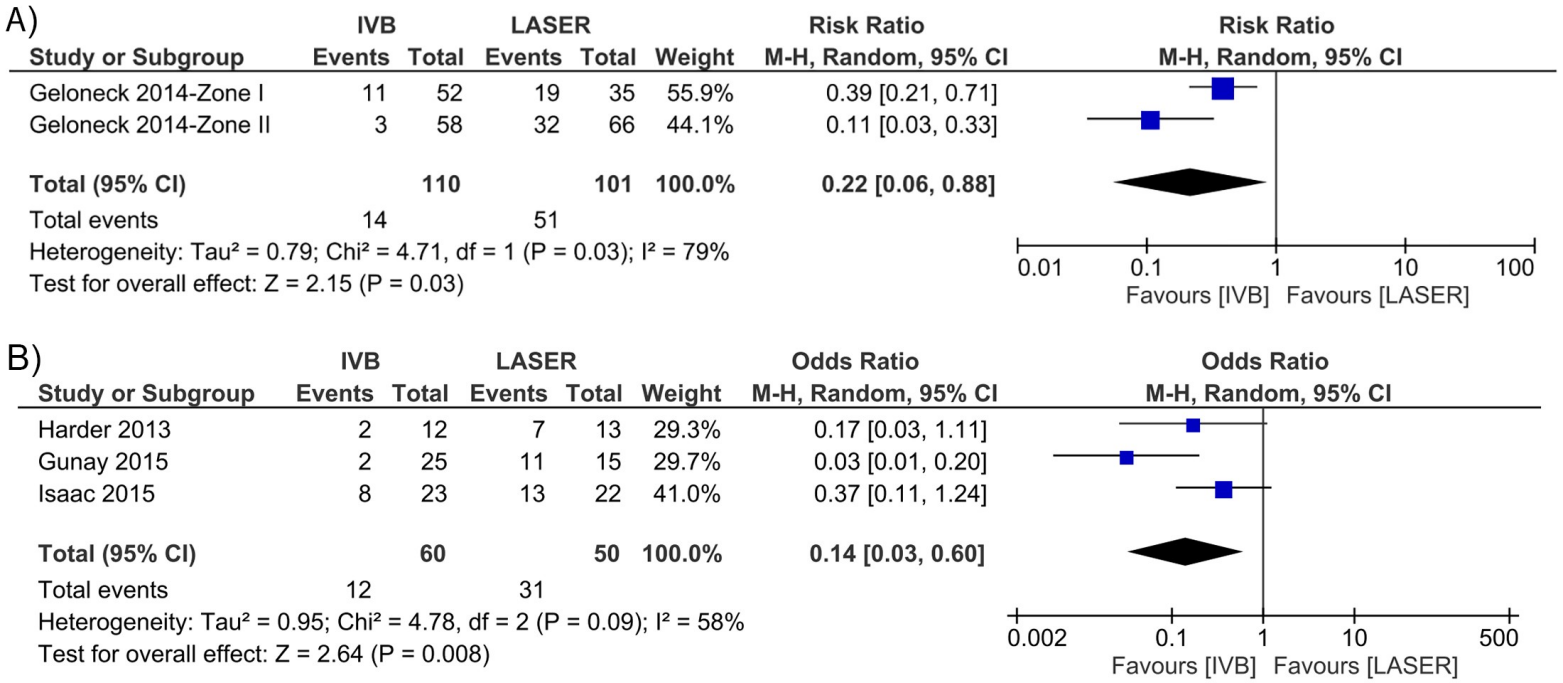
Forest plots of the prevalence of high myopia following IVB and laser treatments. A) Pooled estimates for risk ratios and 95% CI in events of high myopia occurred following two treatments in RCTs. B) Pooled estimates for odds ratios and 95% CI in events of high myopia occurred following two treatments in NRSs. The black diamond symbol on the bottom row shows the estimated true effect size.

Prevalence of high myopia reported in literature varies from 8% [22] to 35% [11]. Data for prevalence of high myopia from 1 RCT (Geloneck 2014 [44]) and 3 NRSs (Harder 2013 [8]; Gunay 2015 [22]; Isaac 2015 [11]) were available for Meta-analysis. A statistically significant lower prevalence of high myopia was shown in IVB treatment compared to laser treatment in both RCT (**Fig 4A**) and NRS (**Fig 4B**) studies (RCT: risk ratio = 0.22; 95% CI, 0.06 to 0.88; *P* = 0.03; NRS: odds ratio = 0.14; 95% CI, 0.03 to 0.6; *P* = 0.008), with significant heterogeneities in both RCT (*I*^*2*^ = 79%, *P* = 0.03) and NRS (*I*^*2*^ = 58%, *P* = 0.09). The meta-analysis of RCT consists of 2 pairs of data by ROP zones from the same study [44], which explains the presence of heterogeneity. Since both Zone I and Zone II data demonstrated consistently significant effects, heterogeneity would not affect our conclusion. For NRSs, by omitting the Gunay et al. study, heterogeneity was reduced from 58% to 0% and the overall effect was not inversed, indicating robust meta-analysis result.

### Magnitude of astigmatism in children treated with IVB versus those treated with laser

**Fig 5:**
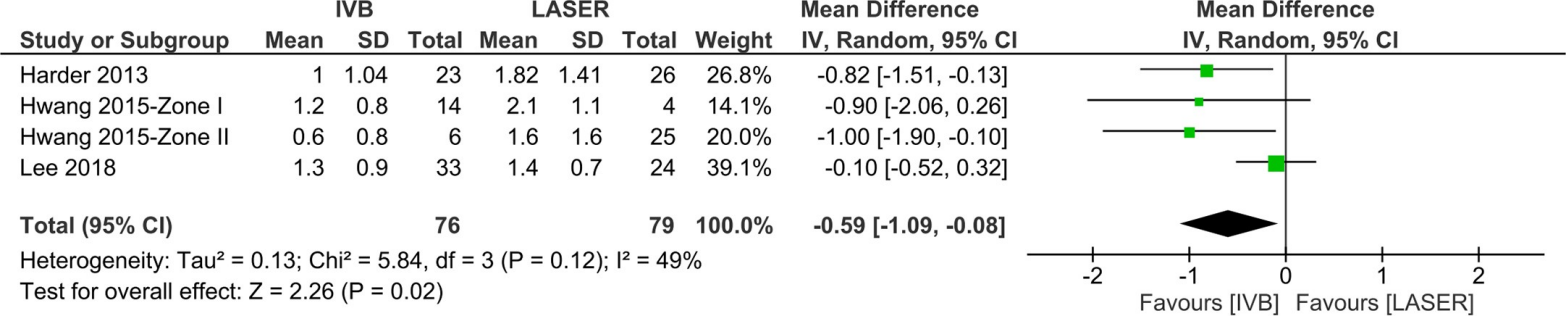
Forest plot of the comparison of astigmatism in cylinder power between IVB and laser treatments. Pooled estimates for mean differences and 95% CI in diopters between two treatments. The black diamond symbol on the bottom row shows the estimated true effect size.

From the 7 studies, data of astigmatism from 3 NRSs (Harder 2013 [8]; Hwang 2015 [10]; Lee 2018 [46]) were available for Meta-analysis. A total of 76 eyes treated by IVB and 79 eyes treated by laser were included. One of the studies compared children with Zone I and Zone II ROP classifications [10]. Therefore, 4 pairs of data were eventually pooled. The mean cylindrical power ranged from 0.6 ± 0.8D to 1.3 ± 0.9D in IVB treatment and 1.4 ± 0.7D to 2.1 ± 1.1D in laser treatment. A statistically significant lower astigmatism was shown in IVB treatment compared to laser treatment (mean difference = -0.59D; 95% CI, -1.09 to -0.08; *P* = 0.02), with low heterogeneity (*I*^*2*^ = 49%, *P* = 0.12). (**Fig 5**)

### Research on longitudinal development of SEQ in IVB-treated individuals are rare

**Fig 6:**
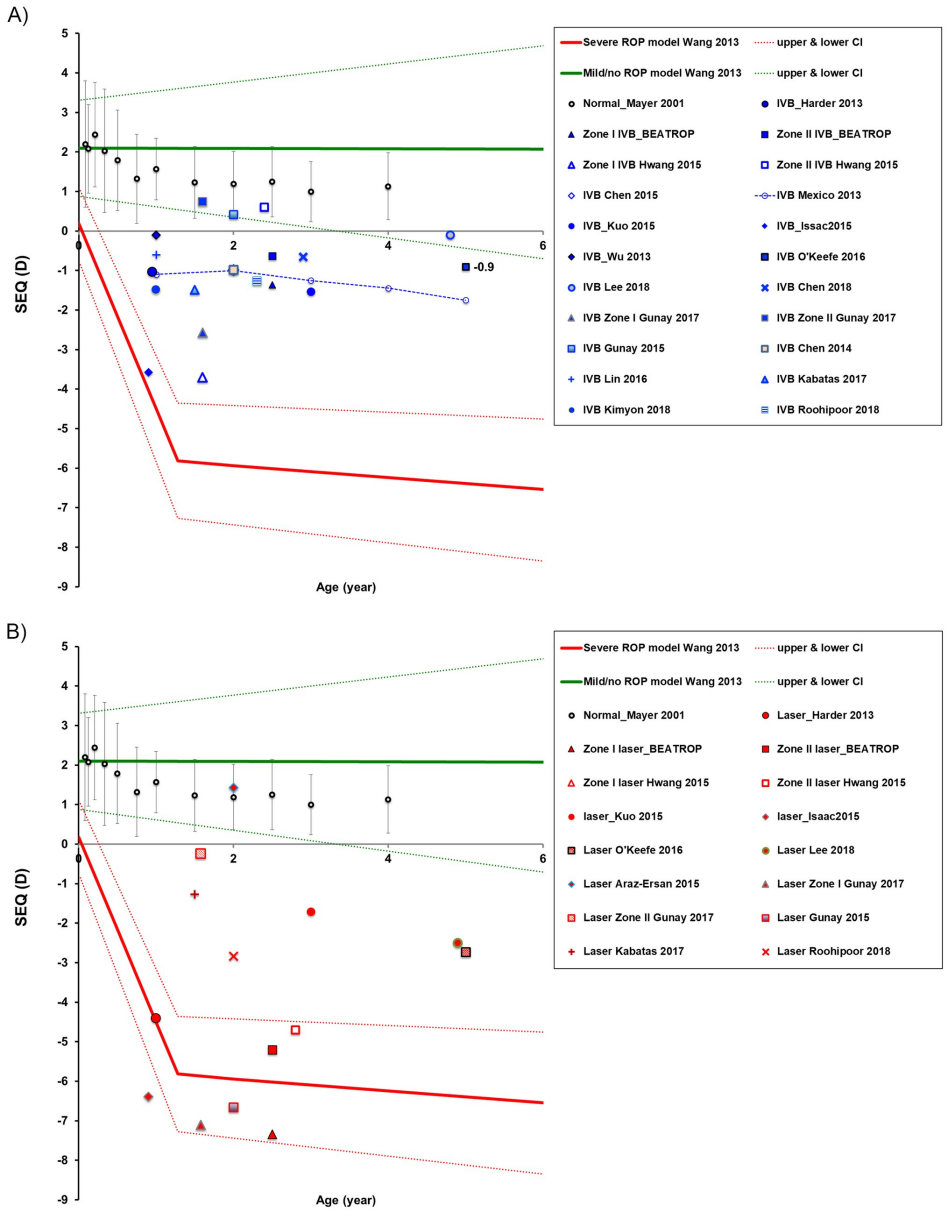
SEQ as a function of age summarized in literature. A) Data from post IVB children. B) Data from the post-laser children. The blue fill color is IVB treatment related, while the red fill color is laser treatment related. The black round circles showed the normal full term population [48]. When information related to Zone I and Zone II, the triangle symbol is for Zone I; the square symbol is for Zone II. The blue symbols with dashed line showed the only longitudinal IVB study from Mexico colleagues [16]. Note: Standard deviations and ranges are not plotted on the figure because standard deviation ranges are generally significantly larger for IVB treated children than the normal population.

In order to visualize the reported data longitudinally, **Fig 6** summarized SEQ in children treated with IVB (**Fig 6A**) or laser (**Fig 6B**) over time, and cross-sectional data from various studies were plotted and overlapped with our previous longitudinal models on children treated with laser photocoagulation [5]. In addition, these data are compared with those from full-term normal children [48]. Obviously, studies on longitudinal SEQ from the post-IVB individuals are very rare. Mexico colleagues followed their patients treated with IVB up to 5 years; however, their patients had higher gestational age (27 to 32 weeks) and birth weight (850 to 1600 grams) than criteria we listed in this study [16].

According to data we listed in **Fig 6A**, the post-IVB children indicated with blue fill symbols demonstrated a low myopic trend compared with the full-term normal population. In **Fig 6B,** the post-laser children indicated with red fill symbols in most studies fall on our model of severe ROP with laser treatment; a few of them are out of the model and still myopic except for an outlier [19].

On average, the SEQ of IVB-treated children falls into the range between laser-treated severe ROP and that of the mild ROP group. Sometimes, high myopia still occurs in IVB-treated children, especially in those with Zone I ROP.

## Discussion

Our meta-analysis and review enable us to visualize refractive error development in IVB-treated preterm children more thoroughly. We found in IVB-treated preterm children: 1) a significantly less myopic refractive error compared with those laser-treated children; 2) high myopia is less prevalent compared with those treated with laser; 3) astigmatism is less severe than in laser-treated children, although it remains significant in both groups; and, 4) although significantly less myopic than laser-treated children even across age, SEQ development of most IVB-treated children is still relatively abnormal compared with the normal full-term children.

### Prevalence, magnitude and range of myopia in children treated with IVB

In most of the published studies, SEQ was used to describe refractive error, thus our discussion of myopia development is limited largely to SEQ. According to existing literature, refractive error in IVB-treated children demonstrates the following characteristics:

1. Prevalence of myopia after IVB treatment remains high. Although less prevalent than laser-treated eyes, all studies reported that some IVB-treated children developed myopia.
2. Compared with children treated with laser photocoagulation, post-IVB children on average have a significantly lower SEQ or are less myopic [7, 8, 10, 22]. For example, Harder et al. reported that the mean SEQ in IVB-treated preterm infants was -1.04 ± 4.24D, which is considerably less myopic than -4.41 ± 5.50D in the laser-treated group [8]. On the other hand, two studies reported there is no statistically significant difference in refractive error between IVB and laser groups [11, 14]. However, one of these studies suggested that increased laser ablation spots might induce more severe myopia. The number of laser spots applied in the study (515 ± 130 spots) was significantly lower than that in the BEAT-ROP study (1954 ± 1288 spots). It was proposed that myopia increased by -0.14 ± 0.05D for every 100 laser-application spots [14].
3. In most studies, the range of refractive error in post-IVB infants varies largely from hyperopia to myopia. A SEQ range of as large as -15D to 6.5D was reported in studies with lower gestational age (mean < 27 weeks). Harder et al. reported the post-IVB refraction had a range of -12.5D to 4.63D [8]; BEAT-ROP reported the IVB group ranged from -13D to 6D [7]; the Hwang et al. study ranged from -8.9D to 2.5D [10]; Chen et al. ranged from -15.6D to 5.5D [13]; and the Isaac et al. study ranged from -15D to 6.5D [11]. By contrast, smaller ranges of refractive error were reported in studies with higher gestational age (mean > 27 weeks). Refractive error ranged from -6.75D to 2.5D in a Mexican study [16]; Kuo et al. reported that no high myopia occurred in their study, and their refractive data still ranged from -5.9D to 1.5D [14]. Note, with such a large range of refractive error, refraction could vary according to individuals. A case report described refractive error in triplets: one of the preterm triplets treated with IVB developed high myopia (-9.75D), while the other two infants had mild hyperopia [49]. With the same gestational age, three infants demonstrated various refractions. Therefore, longitudinal post-IVB data is needed to identify risk factors for myopia and high myopia and to guide the development of preferred practice patterns for monitoring cycloplegic refractions and early optical correction. Further longitudinal analysis based on zones in IVB-treated patients may reveal more information.
4. Posterior ROP zone results in more severe myopia. BEAT-ROP reported that eyes with Zone I ROP are significantly more myopic compared with eyes with Zone II ROP, while there was no difference for either the IVB arm or laser arm. Hwang et al. separated refractive error according to zone. Notably, in the IVB group, eyes with Zone I ROP were more myopic than eyes with Zone II ROP. In addition, Gunay et al. reported that SEQ with Zone I ROP was significantly more myopic than that with Zone II ROP for both IVB and laser groups [23]. Larranaga-Fragoso reported that the mean SEQ was 2.00D in their Zone II ROP [50].

### Astigmatism in children treated with IVB

Generally, IVB-treated children still have a higher prevalence of astigmatism than full-term born normal children. The Harder group reported astigmatism is significantly lower in the IVB group (1.00 ± 1.04D of cylinder) than in the laser group (1.82 ± 1.41 of cylinder) [8]. Hwang et al. reported that astigmatism in the IVB group is about half of the magnitude of that in the laser group in both Zone I and Zone II subgroups; however, no significant difference in astigmatism was found between laser-treated and IVB treated children [10].

Chen et al. reported that the magnitude of astigmatism is about 2.23 ± 1.53D in IVB-treated children. They also considered the axis of astigmatism and reported that 85% of patients had with-the-rule astigmatism [12]. Their results are similar to what we previously found in laser-treated patients [5]. At 5-year follow up, Lee et al. reported less astigmatism (1.3 ± 0.9D) but, still, a high proportion (76%) had with-the-rule astigmatism [46]. In summary, astigmatism, mostly with-the-rule astigmatism, remains a significant factor in most children treated with IVB, although it is less severe than in laser-treated children.

### Anisometropia in children treated with IVB is rarely reported

In children with severe ROP treated with a laser, we previously found that anisometropia is prevalent and even increases with age [5]. Anisometropia has not been extensively investigated in children with ROP. In post-IVB children, the majority of papers in **Table 1** reported data combining the right eyes and the left eyes. Kuo et al. reported that refractions were not significantly different between the right and left eyes [14]. Harder et al. separated data from the right eyes and left eyes, and they, interestingly, reported that right eyes were more myopic than the left eyes [9]. The interocular difference suggested that IVB-treated patients might be associated with anisometropia. Two papers by Chen et al. [13, 21] listed individual both-eye data; by interocular difference we can calculate prevalence of anisometropia (defined interocular SEQ difference≥1D and/or interocular cylindrical power difference≥1D) was 7/20 (35%) in IVB-treated children. According to Gunay et al. study, the incidence of refractive anisometropia was significantly higher in the laser-treated group (66.7%) than the IVB group (20%, *P* = 0.009) [22].

In summary, although many studies did not report anisometropia, anisometropia is still a potential issue in a relatively large portion of children treated with IVB. Potential anisometropia at an early age indicates higher risk of amblyopia in this population.

### Limitations

There are some limitations in the present study: 1) due to the nature of treatment for severe ROP, high-quality RCTs are lacking, more robust conclusions could be drawn if more RCTs were included; 2) significant variability was present in gestational age, follow-up ages, and anti-VEGF doses across the included studies, which might affect the refractive results among studies. However, sensitivity analyses demonstrated robust results by omitting those heterogenous studies. Since obvious variability existed in follow-up ages amongst included studies, we did further sensitivity analyses by omitting the two studies that had a notably different follow-up age of 0.9 year (Harder 2013 and Isaac 2015) to check the impact of this variability on our conclusion. Regardless of which of the two studies was omitted, the heterogeneities for SEQ increased and the robustness of the overall effects was not affected. Because these two studies did not appear to be significant causes of the heterogeneity, we omitted their sensitivity analysis descriptions in the manuscript.

### Future directions

We have reviewed the refractive error findings of children treated with anti-VEGF agents for ROP published in studies conducted within the past decade. Several noteworthy points shed light on how to better understand this issue in the future:

1. For IVB-treated patients, severity of ROP (zone and stage) information may be the most critical factors for refractive error development. Longitudinal analysis for individuals based on the zone of ROP in IVB-treated patients should be illuminating. As mentioned by Darlow et al.[51], we still need to investigate the long-term effects of IVB therapy on refractive error development.
2. Astigmatism and anisometropia have not been adequately studied in children with ROP treated with anti-VEGF agents. Instead of simply analyzing additive data from both eyes, it would be more informative if data were analyzed according to individuals and analyzed between eyes.
3. Although Harder et al. reported that treatment with lower dose IVB, 0.375mg, showed high efficacy, so far there is little information related to refractive error outcomes with lower doses of IVB [52]. Very recently, a much lower dosage, at 0.031mg, has been found effective in treating ROP [53, 54], and refractive error development associated such a low dosage could be of interest for the long term.
4. Biometric data, particularly OCT and ultrasonography, for eyes treated with anti-VEGF agents is rare. This data is needed to determine whether the altered anterior segment maturation that causes myopia is associated with severity of ROP or with laser treatment.
5. Mintz-Hittner & Geloneck suggested that there may be a differential effect of specific anti-VEGF agents on refractive error [36]. More recently, IVR has also been used to treat ROP and reported to be an effective treatment of ROP [55–57]. However, very limited refractive error information exists in IVR-treated patients. Moreover, limited literature reported inconsistent conclusions on whether refractive error development differed in infants treated with IVR and those treated with IVB [13, 23, 25, 26]. Other anti-VEGF agents such as aflibercept [58] and conbercept [59, 60] have also been used to treat ROP. Follow-up with these agents was short and were not discussed in this review.

In the final analysis, close monitoring of refractive error outcomes is important in children treated with anti-VEGF agents. A great deal of further study will be required to better elucidate refractive development in children with ROP, whose eyes are treated with these drugs.

## Supporting information

S1 File PRISMA 2009 checklist. (DOC)

S2 File Remarks for RCT study quality assessment-Cochrane risk of bias tool. (PDF)

S3 File Remarks for NRS study quality assessment-Newcastle-Ottawa Scale (NOS). (DOC)

S4 File Funnel plot for publication bias test. (PDF)

S5 File Information for excluded studies. (PDF)
