# Supplementary material for: Development of refractive error in children treated for retinopathy of prematurity with anti-vascular endothelial growth factor (anti-VEGF) agents: A meta-analysis and systematic review
Source: PLoS One. 2019 Dec 2;14(12):e0225643. doi: 10.1371/journal.pone.0225643 (PMC6886775; doi:10.1371/journal.pone.0225643)
Supplement: S2 File — (PDF) [file pone.0225643.s002.pdf]

Characteristics of studies

Characteristics of included studies

Geloneck 2014

**Methods**	RCT
**Participants**	Preterm infants who developed ROP in zone I or zone II posterior as stage 3+ ROP or aggressive posterior ROP (APROP)
**Interventions**	intravitreal bevacizumab (IVB) vs laser treatment
**Outcomes**	Spherical equivalent refractive outcomes and their distribution by ROP zone and treatment
**Notes**	Follow-up of the BEAT-ROP cohort

Risk of bias table

Bias	Authors' judgement	Support for judgement
Random sequence generation (selection bias)	Low risk	Quote: “Each infant was then randomized to receive treatment in both eyes as either intravitreal bevacizumab monotherapy or near confluent laser therapy.”<br>Comment: Probably done, since earlier reports from the same group clearly described detailed use of randomization (Mintz-Hittne et al 2011).
Allocation concealment (selection bias)	Low risk	Quote: “A secure, computer-generated randomization schedule stratified on the basis of zone was maintained by a study-group member who did not participate in enrollment. Treatment assignments were revealed to the investigators only after eligibility for enrollment had been confirmed. ” (Mintz-Hittne et al 2011).<br>Comment: Probably done. Detailed use of allocation concealment was described in the earlier report of the same study.
Blinding of participants and personnel (performance bias)	High risk	Quote: “The study, which was controlled but not masked,”<br>Comment: Not done, clearly specified in the paper.
Blinding of outcome assessment (detection bias)	High risk	Quote: “Unmasked practicing pediatric ophthalmologists performed the cycloplegic retinoscopic refractions.”<br>Comment: Not done, clearly specified in the paper.
Incomplete outcome data (attrition bias)	Low risk	Outcome data of all infants who were randomized were included in the analysis; 16.8% of eligible infants were lost to follow-up at 2.5 years of age.
Selective reporting (reporting bias)	Low risk	All outcomes mentioned in the protocol were reported.

Other bias	High risk	Though the infants were randomized, results for the outcomes were provided for the individual eyes.

O'Keeffe 2016

**Methods**	RCT
**Participants**	Premature babies with zone 1 or posterior zone 2 retinopathy of prematurity (ROP).
**Interventions**	intravitreal Bevacizumab vs laser
**Outcomes**	complications, regression/reactivation of ROP, visual outcome, refractive error and systemic complications.
**Notes**	3 eyes initially treated with bevacizumab that showed recurrence of ROP received laser therapy, while 1 eye in the laser group was treated with bevacizumab for recurrence

Risk of bias table

**Bias**	**Authors' judgement**	**Support for judgement**
Random sequence generation (selection bias)	Unclear risk	not specified
Allocation concealment (selection bias)	Unclear risk	not specified
Blinding of participants and personnel (performance bias)	High risk	Not possible, because 1 eye is randomized to intervention, while the other eye is randomized to laser treatment.
Blinding of outcome assessment (detection bias)	Unclear risk	not specified
Incomplete outcome data (attrition bias)	Low risk	No loss to follow-up.
Selective reporting (reporting bias)	Unclear risk	Not clear if all the outcomes were reported
Other bias	High risk	Because randomization was by eyes rather than patients, the eyes randomized to the laser group would have been exposed to both anti-VEGF agents and lase treatments, resulting in better outcomes if there was significant systemic absorption of anti-VEGF agents.

Footnotes
