# Supplementary material for: Development of refractive error in children treated for retinopathy of prematurity with anti-vascular endothelial growth factor (anti-VEGF) agents: A meta-analysis and systematic review
Source: PLoS One. 2019 Dec 2;14(12):e0225643. doi: 10.1371/journal.pone.0225643 (PMC6886775; doi:10.1371/journal.pone.0225643)
Supplement: S3 File — (DOC) [file pone.0225643.s003.doc]

**NEWCASTLE - OTTAWA QUALITY ASSESSMENT SCALE**

**CASE CONTROL STUDIES**

Note: A study can be awarded a maximum of one star for each numbered item within the Selection and Exposure categories. A maximum of two stars can be given for Comparability.

**Harder 2013 study (8★)**

**Selection**

1) Is the case definition adequate?

**a)** yes, with independent validation (Quote: “The study was a retrospective analysis of clinical data obtained during routine care of the children. The diagnosis of ROP was based on the revised guidelines of the International Committee for the Classification of Retinopathy of Prematurity. The criterion for therapy of ROP was the presence of a threshold disease, which was defined as disease stage with a 50% likelihood of progressing to retinal detachment. Threshold disease was considered to be present when stage 3 ROP was present in either zone I or zone II, with at least 5 continuous or 8 total clock hours of disease, and the presence of plus disease.”)**★**

b) yes, eg record linkage or based on self reports

c) no description

2) Representativeness of the cases

**a)** consecutive or obviously representative series of cases (Quote: “Starting in 2008, we discussed with the parents of all affected children the possibility of an off-label intravitreal injection of bevacizumab as an alternative to conventional standard laser treatment performed according to the international guidelines of the Early Treatment for Retinopathy of Prematurity Cooperative Group. The study group included all infants who consecutively received a single intravitreal bevacizumab.”)**★**

b) potential for selection biases or not stated

3) Selection of Controls

a) community controls **★**

**b)** hospital controls (Quote: “The study group was compared with a control group of children who had previously undergone retinal argon laser therapy of ROP in the same center.”)

c) no description

4) Definition of Controls

**a)** no history of disease (endpoint) (Quote: “The study group included all infants who consecutively received a single intravitreal bevacizumab… The control group included infants who had previously undergone retinal argon laser therapy ...”)**★**

b) no description of source

**Comparability**

1) Comparability of cases and controls on the basis of the design or analysis

**a)** study controls for “quote: control group and study group did not differ significantly in birth weight…”. (Select the most important factor.)**★**

**b)** study controls for any additional factor “quote: control group and study group did not differ significantly in birth weight, gestational age, and length of follow-up”**★** (This criteria could be modified to indicate specific control for a second important factor.)

**Exposure**

1) Ascertainment of exposure

**a)** secure record (eg surgical records) (Quote: “The intravitreal bevacizumab injection was performed under sterile conditions in the pediatric intensive care unit for prematurely born infants. After topical anesthesia of the cornea and conjunctiva by repeated application of oxybuprocaine hydrochloride eye drops and after inducing a slight systemic sedation of the children, the external eye and the surrounding skin were disinfected with a povidoneiodine 5% ophthalmic solution (Betadine; Alcon Inc, Fort Worth, Texas, USA) and the eyes were draped…bevacizumab (Avastin) was intravitreally injected at 1.5 mm posterior to the limbus in the temporal inferior quadrant.”)**★**

b) structured interview where blind to case/control status **★**

c) interview not blinded to case/control status

d) written self report or medical record only

e) no description

2) Same method of ascertainment for cases and controls

**a)** yes (both by medical record)**★**

b) no

3) Non-Response rate

**a)** same rate for both groups (all participants completed the follow-up examination)**★**

b) non respondents described

c) rate different and no designation

**Gunay 2015 study (7★)**

**Selection**

1) Is the case definition adequate?

**a)** yes, with independent validation (Quote: “we performed a retrospective review of the medical records of children who received IVB (Group I) as a single treatment modality for APROP. Further, we reviewed a group comprising infants who underwent laser treatment for APROP, designated group II. The diagnosis of APROP was made according to the “The International Classification of ROP.”)**★**

b) yes, eg record linkage or based on self reports

c) no description

2) Representativeness of the cases

a) consecutive or obviously representative series of cases**★**

**b)** potential for selection biases or not stated

3) Selection of Controls

a) community controls **★**

b) hospital controls

**c)** no description

4) Definition of Controls

**a)** no history of disease (endpoint) (Quote: “children who received IVB (Group I) as a single treatment modality for APROP. Further, we reviewed a group comprising infants who underwent laser treatment for APROP, designated group II.”)**★**

b) no description of source

**Comparability**

1) Comparability of cases and controls on the basis of the design or analysis

**a)** study controls for “quote: No significant differences in gestational age (GA), birth weight (BW), or gender were observed between group I and group II”. (Select the most important factor.)**★**

**b)** study controls for any additional factor “quote: No significant differences in gestational age (GA), birth weight (BW), or gender were observed between group I and group II”**★** (This criteria could be modified to indicate specific control for a second important factor.)

**Exposure**

1) Ascertainment of exposure

**a)** secure record (eg surgical records) (Quote: “All injections were performed at an office-based injection clinic. Following topical anesthesia …0.625 mg bevacizumab...; Infants in group II were treated with laser ablation according to the methods of the Early Treatment for ROP (ETROP) study. Following topical anesthesia…”)**★**

b) structured interview where blind to case/control status **★**

c) interview not blinded to case/control status

d) written self report or medical record only

e) no description

2) Same method of ascertainment for cases and controls

**a)** yes (both by medical record)**★**

b) no

3) Non-Response rate

**a)** same rate for both groups (all participants completed the follow-up examination)**★**

b) non respondents described

c) rate different and no designation

**Hwang 2015 study (8★)**

**Selection**

1) Is the case definition adequate?

**a)** yes, with independent validation (Quote: “Infants whose ROP met the criteria for type 1 ROP, as defined by the Early Treatment for Retinopathy of Prematurity study as zone I with any stage with plus disease, zone I with stage 3 without plus disease, and zone II with stage 2 or 3 with plus disease, were treated”)**★**

b) yes, eg record linkage or based on self reports

c) no description

2) Representativeness of the cases

**a)** consecutive or obviously representative series of cases (Quote: “Included in the study were consecutive infants with type 1 ROP who received either IVB or PRP between …; The data from infants treated with either IVB or PRP for type 1 ROP between 2008 and 2012 were recorded from 2 medical centers in Atlanta, Georgia.”)**★**

b) potential for selection biases or not stated

3) Selection of Controls

**a)** community controls (Quote: “…received either IVB or PRP between January 2008 and December 2012 at Children’s Healthcare of Atlanta at Egleston Hospital and Emory Midtown Hospital in Atlanta, Georgia”, multi-center)**★**

b) hospital controls

c) no description

4) Definition of Controls

**a)** no history of disease (endpoint) (Quote: “Included in the study were consecutive infants with type 1 ROP who received either IVB or PRP”)**★**

b) no description of source

**Comparability**

1) Comparability of cases and controls on the basis of the design or analysis

**a)** study controls for “mean birth age between the study group and control group was not significantly different (as described in the demographic table)”. (Select the most important factor.)**★**

**b)** study controls for any additional factor “birth weight, gender and maternal race were not significantly different between study group and control group (as described in the demographic table)”**★** (This criteria could be modified to indicate specific control for a second important factor.)

**Exposure**

1) Ascertainment of exposure

**a)** secure record (eg surgical records) (Quote: “An injection of bevacizumab 0.625 mg (0.025 ml solution) was performed through the pars plicata using.... For PRP…An indirect laser at wavelength 810 nm then was used to apply photocoagulation …”)**★**

b) structured interview where blind to case/control status **★**

c) interview not blinded to case/control status

d) written self report or medical record only

e) no description

2) Same method of ascertainment for cases and controls

**a)** yes (both by medical record)**★**

b) no

3) Non-Response rate

a) same rate for both groups **★**

**b)** non respondents described (Quote: “Refractive error data were available for 49 (93%) of 54 eyes from 26 (93%) of 28 patients (Table 3). Twenty eyes in 10 patients were treated with IVB… Twentynine eyes in 16 patients were treated with PRP...”)

c) rate different and no designation

**Isaac 2015 study (7★)**

**Selection**

1) Is the case definition adequate?

**a)** yes, with independent validation (Quote: “…diagnosis of zone I ROP according to the International Classification of Retinopathy ofPrematurity (ICROP)... Two ophthalmologists reviewed fundus images (RetCam; Clarity Medical Systems, Pleasanton, CA) taken immediately before treatment to confirm the accuracy... Zone II posterior was defined as in the BEAT-ROP study as a circle with a radius ...”)**★**

b) yes, eg record linkage or based on self reports

c) no description

2) Representativeness of the cases

**a)** consecutive or obviously representative series of cases (Quote: “The medical records of all infants treated for type 1 ROP from January 2009 until May 2013 at the Hospital for Sick Children, Toronto, were retrospectively reviewed.”)**★**

b) potential for selection biases or not stated

3) Selection of Controls

a) community controls **★**

**b)** hospital controls (Quote: “…The medical records of infants treated with IVB or laser photocoagulation at our institution from January 2009 to May 2013 were retrospectively reviewed”)

c) no description

4) Definition of Controls

**a)** no history of disease (endpoint) (Quote: “Infants treated with intravitreal injection of bevacizumab or with retinal laser photocoagulation using...”)**★**

b) no description of source

**Comparability**

1) Comparability of cases and controls on the basis of the design or analysis

**a)** study controls for “mean gestational age between the study group and control group was not significantly different (as described in the demographic table)”. (Select the most important factor.)**★**

**b)** study controls for any additional factor “birth weight, gender and postmenstrual age were not significantly different between study group and control group (as described in the demographic table)”**★** (This criteria could be modified to indicate specific control for a second important factor.)

**Exposure**

1) Ascertainment of exposure

**a)** secure record (eg surgical records) (Quote: “Bevacizumab 0.625 mg/0.025 ml was injected into the vitreous cavity with a 30-gauge needle 1.0 mm posterior to …”)**★**

b) structured interview where blind to case/control status **★**

c) interview not blinded to case/control status

d) written self report or medical record only

e) no description

2) Same method of ascertainment for cases and controls

**a)** yes (both by medical record)**★**

b) no

3) Non-Response rate

a) same rate for both groups **★**

**b)** non respondents described (Quote: “All 12 infants in the laser group were treated in both eyes. However, in 2 infants that had disease in zone II posterior, the other eye was treated for zone II posterior stage 3 preplus (type 2 ROP), and thus these two eyes were excluded from the analysis....”)

c) rate different and no designation

**Lee 2018 study (7★)**

**Selection**

1) Is the case definition adequate?

**a)** yes, with independent validation (Quote: “…The ROP grade was determined by the maximal severity in the acute stage according to the International Classification of ROP criteria. The indications for treatment depended on the development of type I disease as defined by a previous ET-ROP study...”)**★**

b) yes, eg record linkage or based on self reports

c) no description

2) Representativeness of the cases

a) consecutive or obviously representative series of cases **★**

**b)** potential for selection biases or not stated

3) Selection of Controls

a) community controls **★**

**b)** hospital controls (Quote: “…This study recruited 3 groups of preschool-aged children with histories of treated type I ROP who were followed at our hospital...”)

c) no description

4) Definition of Controls

**a)** no history of disease (endpoint) (Quote: “The first group included children who had received IVB monotherapy only; the second group included children who had received conventional laser photocoagulation only...”)**★**

b) no description of source

**Comparability**

1) Comparability of cases and controls on the basis of the design or analysis

**a)** study controls for “mean gestational age between the study group and control group was similar (26.6 ± 1.6 weeks vs 26.6 ± 1.5 weeks, as described in the demographic table)”. (Select the most important factor.)**★**

**b)** study controls for any additional factor “Significant differences were not observed in mean birth weight and the age at the time of examination between groups”**★** (This criteria could be modified to indicate specific control for a second important factor.)

**Exposure**

1) Ascertainment of exposure

**a)** secure record (eg surgical records) (Quote: “In the IVB group, 0.625 mg (0.025 mL) of bevacizumab (Avastin; Genentech Inc, San Francisco, California, USA) was injected via the pars plicata. In the conventional laser group, near confluent 810-nm diode laser photocoagulation was performed on the entire area ...”)**★**

b) structured interview where blind to case/control status **★**

c) interview not blinded to case/control status

d) written self report or medical record only

e) no description

2) Same method of ascertainment for cases and controls

**a)** yes (both by medical record)**★**

b) no

3) Non-Response rate

**a)** same rate for both groups (all participants completed the follow-up examination)**★**

b) non respondents described

c) rate different and no designation
