# Supplementary figures and images for: Development of refractive error in children treated for retinopathy of prematurity with anti-vascular endothelial growth factor (anti-VEGF) agents: A meta-analysis and systematic review
Source: PLoS One. 2019 Dec 2;14(12):e0225643. doi: 10.1371/journal.pone.0225643 (PMC6886775; doi:10.1371/journal.pone.0225643)

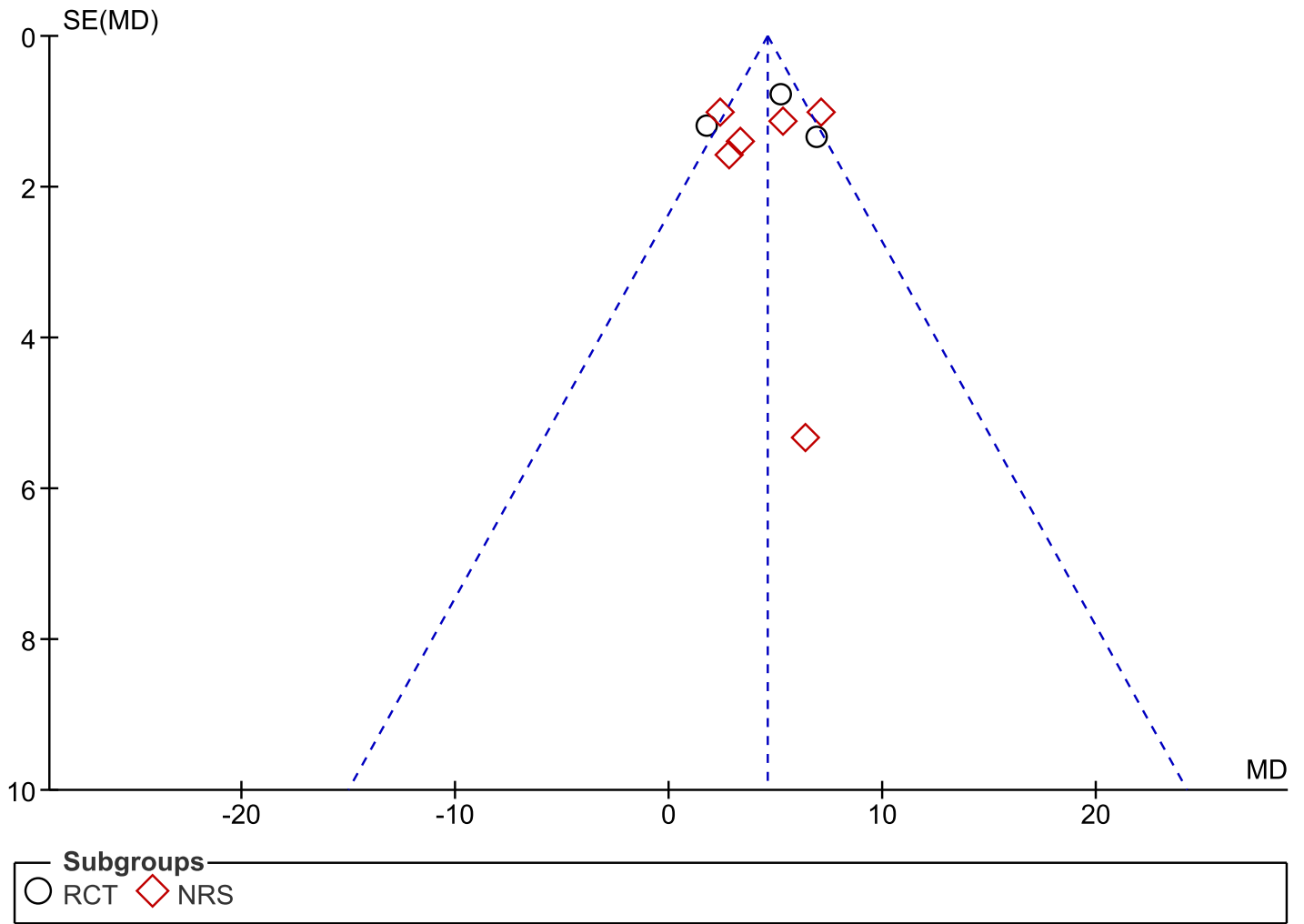

Supplement: S4 File — (PDF) [file pone.0225643.s004.pdf]
