# Supplementary material for: Development of refractive error in children treated for retinopathy of prematurity with anti-vascular endothelial growth factor (anti-VEGF) agents: A meta-analysis and systematic review
Source: PLoS One. 2019 Dec 2;14(12):e0225643. doi: 10.1371/journal.pone.0225643 (PMC6886775; doi:10.1371/journal.pone.0225643)
Supplement: S5 File — (PDF) [file pone.0225643.s005.pdf]

Characteristics of studies

Characteristics of excluded studies

Harder 2012

**Reason for exclusion**	A research letter, not the complete research report.

Kuo 2015

**Reason for exclusion**	Failed to meet the selection criteria. Some ROP children's birthweights were more than 1500 grams and gestational ages more than 30 weeks.

Gunay 2017

**Reason for exclusion**	Failed to meet the selection criteria. Some ROP children's birthweights were more than 1500 grams and gestational ages more than 30 weeks.

Kabatas 2017

**Reason for exclusion**	Failed to meet the selection criteria. Some ROP children's birthweights were more than 1500 grams and gestational ages more than 30 weeks, and the baseline birthweights were uneven between the treatments.

Roohipoor 2018

**Reason for exclusion**	Failed to meet the selection criteria. Some ROP children's birthweights were more than 1500 grams and gestational ages more than 30 weeks.

Kang 2019

**Reason for exclusion**	Failed to meet the selection criteria. Some ROP children's gestational ages were more than 30 weeks, and the baseline gestational ages between treatments were significantly uneven.

Footnotes
